# Redox Regulation of PTEN by Reactive Oxygen Species: Its Role in Physiological Processes

**DOI:** 10.3390/antiox13020199

**Published:** 2024-02-04

**Authors:** Vu Hoang Trinh, Thang Nguyen Huu, Dhiraj Kumar Sah, Jin Myung Choi, Hyun Joong Yoon, Sang Chul Park, Yu Seok Jung, Seung-Rock Lee

**Affiliations:** 1 Department of Biochemistry, Department of Biomedical Sciences, Chonnam National University Medical School, Gwangju 501190, Republic of Korea; trinhhoangvu@jnu.ac.kr (V.H.T.); 206847@jnu.ac.kr (T.N.H.); 197784@chonnam.edu (D.K.S.); choijm2@jnu.ac.kr (J.M.C.); hjms0320@jnu.ac.kr (H.J.Y.); 2 Department of Oncology, Department of Medical Sciences, Pham Ngoc Thach University of Medicine, Ho Chi Minh City 700000, Vietnam; 3 The Future Life & Society Research Center, Advanced Institute of Aging Science, Chonnam National University, Gwangju 61469, Republic of Korea; parksc@snu.ac.kr; 4 Chonnam National University Medical School, Gwangju 501190, Republic of Korea; william67@jnu.ac.kr

**Keywords:** PTEN, redox regulation, oxidative inhibition, ROS, cell signaling

## Abstract

Phosphatase and tensin homolog (PTEN) is a tumor suppressor due to its ability to regulate cell survival, growth, and proliferation by downregulating the PI3K/AKT signaling pathway. In addition, PTEN plays an essential role in other physiological events associated with cell growth demands, such as ischemia-reperfusion, nerve injury, and immune responsiveness. Therefore, recently, PTEN inhibition has emerged as a potential therapeutic intervention in these situations. Increasing evidence demonstrates that reactive oxygen species (ROS), especially hydrogen peroxide (H_2_O_2_), are produced and required for the signaling in many important cellular processes under such physiological conditions. ROS have been shown to oxidize PTEN at the cysteine residue of its active site, consequently inhibiting its function. Herein, we provide an overview of studies that highlight the role of the oxidative inhibition of PTEN in physiological processes.

## 1. Introduction

Phosphatase and tensin homolog (PTEN) belongs to the protein tyrosine phosphatase (PTP) family and was initially identified as a tumor suppressor with a specific role in regulating cell growth. The structure of human PTEN consists of an N-terminal-phosphatidylinositol (4,5)-bisphosphate (PIP2)-binding/phosphatase catalytic domain followed by a C2-lipid-binding domain, which enables its membrane-associated function, a C-terminal tail domain, and a PDZ-binding domain. The distinctive phosphatase function feature of PTEN, in comparison with other PTPs, is counteracting the activity of class I phosphoinositide 3-kinases (PI3Ks) through the dephosphorylation of phosphatidylinositol-3,4,5-triphosphate (PIP3) to PIP2 [1,2,3,4]. Via this mechanism, PTEN acts as a suppressor of the phosphoinositide 3-kinases/protein kinase B (PI3K/AKT) pathway. Since the PI3K/AKT signaling pathway promotes protein synthesis, cell survival, proliferation, and migration [5,6], PTEN dysfunction can contribute to the development of certain hereditary tumorigenesis disorders such as Cowden syndrome, Proteus syndrome, Bannayan–Riley–Ruvalcaba syndrome, and Lhermitte–Duclos disease [7], as well as various cancers including breast [8], thyroid [9], endometrium [10], prostate [11], brain [12], and skin cancer [13].

PTEN expression can be regulated via genetic, epigenetic, post-transcriptional, and post-translational mechanisms that influence the PTEN gene, mRNA, and protein [14]. Epigenetic PTEN silencing involves gene promoter methylation and histone modification. At the post-transcriptional level, microRNAs have been well studied for their capacity to inhibit PTEN expression, especially in cancers. Kinases such as glycogen synthase kinase GSK3, casein kinase CK2, and serine–threonine kinase STK11 can inactivate PTEN by phosphorylating serine and threonine residues in the C-terminal tail region [14,15]. Since an elevated PI3K/AKT signaling pathway has been demonstrated to be beneficial in physiological processes that require cell regeneration, inhibiting PTEN, a negative regulator of this pathway, has been considered a prospective therapy for neurodegenerative diseases, ischemia, infection, and insulin-resistant metabolic disorders [14]. In studies about the therapeutic modalities for those circumstances, biperoxovanadium compounds have been extensively used as specific PTEN inhibitors [5]. Additionally, the interplay between miRNAs and PTEN is also implicated in the oxidative-stress-induced pathogenesis of those non-malignant diseases; thus, utilizing miRNAs as PTEN regulators, such as miR302-367 [16], miR-217 [17], miR-29a [18], and miR-22 [19], can yield a therapeutic approach [20]. 

Like other members of the PTP family that contain a cysteine residue in their active site, PTEN can undergo oxidative inactivation by reactive oxygen species (ROS) [21]. ROS are generated via endogenous sources such as NADPH oxidase (NOX), nitric oxide synthase (NOS), xanthine oxidase, aldehyde oxidase, cyclo-oxygenase, cytochrome P450 2E1, and electron leakage from mitochondria, as well as exogenous sources such as smoke, ultraviolet light, radiation, and drugs [22,23]. Superoxide (O_2_^•−^) can react with nitric oxide (NO) to form peroxynitrite (ONOO^−^) or be transformed into hydrogen peroxide (H_2_O_2_) by superoxide dismutase (SOD), vitamin E, or vitamin C. Oxidative inactivation of PTEN, which can serve as a physiological regulatory mechanism, is executed by ROS not only via oxidative stress but also via cellular signaling transductions, for example, growth-factor-stimulation-derived NOXs [24]. A growing body of evidence has indicated that ROS are produced and utilized in physiological circumstances to function as significant signaling messengers, facilitating the coordination of various fundamental processes, including inflammation, survival, proliferation, differentiation, apoptosis, signal transduction, and other critical events [25,26,27,28,29]. Oxidative stress can occur during chronic low-grade systemic inflammation, in which pro-inflammation cytokines secreted from senescent cells induce the production of ROS, consequently leading to the oxidation of cellular components [30]. 

The ROS that have such cellular physiological functions are predominantly generated in the cell’s plasma membrane and endomembrane via the activity of NOXs [31]. H_2_O_2_ is the major ROS responsible for initiating redox-dependent signaling within the cell’s cytosol [32], and the source of this physiological H_2_O_2_ is also related to the activities of membrane-associated NOX complex and specialized cells such as phagocytes [33,34]. Lee et al. were the first to demonstrate the reversible inactivation of PTEN by H_2_O_2_. During this process, the Cys124 catalytic residue in the active site of PTEN is oxidized and forms a disulfide bond with Cys71, thus being inactivated. This inactivation is reversible because oxidized PTEN is persistently reduced back to its active form by the redox homeostasis systems, particularly the thioredoxin (Trx) system, which is ubiquitous in the cellular environment [35,36]. In mammalian cells, there are abundant antioxidants, such as Trxs, glutathione (GSH), glutaredoxins (Grx), and peroxiredoxins (Prx). The Trx system, which is composed of thioredoxin reductase (TrxR) and NADPH, can act as an electron donor to a variety of enzymes, including PTEN, and catalyze the reduction of disulfide bonds [37]. The Prx, GSH, and Grx systems also engage in the reduction of oxidized PTEN, thereby contributing to the redox regulation of PTEN [38,39,40]. Prx can scavenge H_2_O_2_ at a fast speed. Under mild oxidative stress conditions, Prx I not only protects PTEN from oxidation but also enhances its activity via direct interaction [41,42]. Notably, the oxidative inhibition of PTEN by H_2_O_2_ has been experimentally demonstrated to increase the PI3K/AKT signaling pathway [43]. 

Peroxynitrite (ONOO^−^) can also oxidize cysteine residues within PTPs, leading to oxidative inhibition. This process might be considerably faster and more effective in inactivating PTPs at lower concentrations than H_2_O_2_. This suggests that peroxynitrite may be the primary biological mediator responsible for PTPs’ inactivation, consequently enhancing tyrosine phosphorylation in situations related to oxidative stress [44]. However, the impact of peroxynitrite on phosphotyrosine-dependent signaling can manifest as either activation or inhibition. The upregulation of this signaling could arise from PTPs’ inactivation by a low concentration of peroxynitrite, and this feature has typical characteristics of cell signaling, being transient and reversible. Nevertheless, how peroxynitrite affects the PI3K/AKT pathway is still controversial [45].

The oxidative inactivation of PTEN leads to an increase in PI3K/AKT downstream signaling, which subsequently induces its physiological effects [43,46,47]. Recently, bicarbonate/carbon dioxide (HCO_3_^−^/CO_2_) has emerged as a pivotal factor in promoting the oxidative reactivity of H_2_O_2_ by creating a higher reactive form called peroxymonocarbonate (HCO_4_^−^) [48,49,50]. Since there are several meticulous and comprehensive reviews about the regulators of PTEN and their impacts on the PI3K/AKT signaling pathway, as well as their implications in physiology and diseases, we focus on the role of the oxidative inhibition of PTEN in physiological processes. In addition, we also mention the role of bicarbonate/carbon dioxide in the oxidation of PTPs by H_2_O_2_.

## 2. Oxidative Inhibition of PTEN by ROS in Physiological Processes 

### 2.1. Cardiovascular Remodeling

Studies indicate the involvement of the serine/threonine kinase AKT as a mediator in the process of ischemic preconditioning, a short transient period of sustenance during ischemia-reperfusion injury [51,52,53,54]. In ischemic preconditioning, AKT signaling is upregulated and prevents cardiomyocytes from undergoing apoptosis [53,54,55,56]. The PI3K/AKT/mTOR pathway plays a significant role in protecting against ischemia-reperfusion injury, particularly in the context of ischemic preconditioning in cardiac tissue. Accordingly, reversible PTEN downregulation has been suggested as a viable therapeutic approach to mitigate ischemia-reperfusion-related cardiac damage [57]. A study revealed that PTEN plays a pivotal role in the post-myocardial infarction remodeling process: Partial PTEN inactivation, by regulating the AKT signaling pathway, can increase interleukin IL-10 and consequently decrease tumor necrosis factor TNFα and matrix metalloproteinase MMP2 expression in the heart. However, the authors were not able to determine the exact source of generated IL-10, apart from immune cells. It probably comes from endothelial cells and fibroblasts [58]. Several research studies demonstrate that IL-10 can eventually attenuate apoptosis and facilitate cardiac remodeling after myocardial infarction [59,60,61,62]. Hence, PTEN inhibition could be an effective approach for improving cardiac conservation after ischemia [63,64].

During acute myocardial infarction, the heart suffers from oxidative stress with increased ROS levels [23]. In the acute and chronic cellular response to this event, NOX2 is overexpressed in human cardiomyocytes, which may not interfere with the activity of macrophages [65,66,67]. Since PTEN oxidation is likely to occur near the site of ROS formation and both PIP3 and the NOX complex are located in the plasma membrane, H_2_O_2_ generated from NOXs is the primary candidate for inhibiting the PI3K/AKT pathway via PTEN oxidation. There is substantial supporting evidence indicating that elevated PIP3 signaling contributes to the activation of the NOX complex in both phagocytic and non-phagocytic cells. The increase in PIP3 levels is proposed to be a key factor in initiating the activation of the NOX complex [41,43]. This may create a circular impact, where ROS generated from NOXs can inhibit PTEN and enhance the PI3K/AKT pathway, which, in turn, promotes NOX activity.

**Figure 1:**
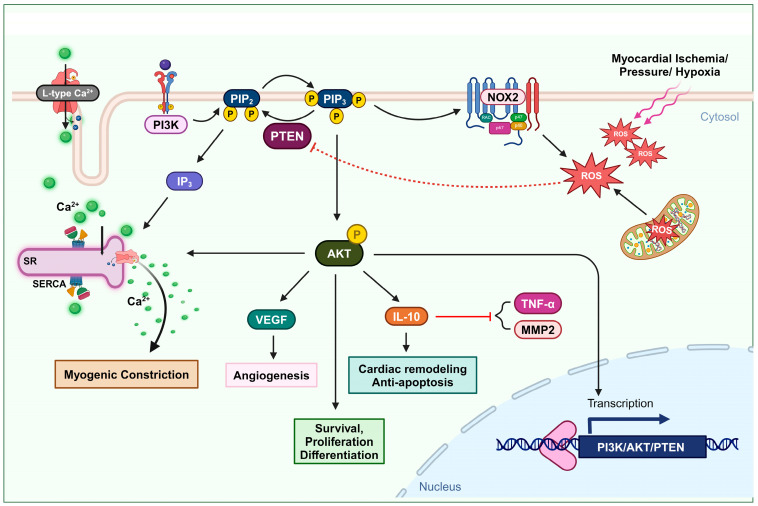
Oxidation of PTEN in cardiovascular remodeling and myogenic constriction. Ischemia or elevated blood pressure conditions induce the production of ROS. These ROS deactivate PTEN, leading to an increase in the AKT signaling pathway. The activation of the AKT pathway enhances cell survival, proliferation, and differentiation. Furthermore, PTEN-mediated AKT activation upregulates IL-10 expression, promoting cardiac remodeling and preventing apoptosis. It also elevates VEGF expression, facilitating angiogenesis. This mechanism also involves L-type calcium channel activity and the formation of IP3, which stimulates Ca^2+^ secretion, thus increasing intracellular Ca^2+^ levels and promoting myogenic constriction.

Cai and Semenza were the first to describe the modulation of PTEN during ischemia-reperfusion injury. During the first 15 min of ischemia, PTEN undergoes dephosphorylation and proteasomal degradation. However, the kinetics reveal that not all PTEN activity is impaired during this initial phase and AKT phosphorylation increases without any significant changes. This indicates that the dephosphorylation and degradation of PTEN do not greatly hinder its function. However, in the subsequent initial phase of reperfusion, there is a notable increase in oxidized PTEN and, consequently, phosphorylated AKT. Their findings clarify that the surge in AKT phosphorylation during this short reperfusion period is caused by the oxidative inhibition of the remaining PTEN [68]. Simultaneously, elevated levels of ROS have been observed in both injured cardiomyocytes and intact hearts during ischemia-reperfusion events [68,69]. Therefore, the oxidation of PTEN during the initial reperfusion period is related to the concurrent rise in ROS levels. (Figure 1).

One vital mechanism of injured tissue in cases of blood supply shortage, due to ischemia or infarction events, is angiogenesis. Angiogenesis is defined as the formation of new blood vessels [70]. Vascular endothelial growth factor (VEGF) is associated with promoting angiogenesis. Upregulation of VEGF can be a potential treatment approach to induce axonal outgrowth and following angiogenesis after cerebral ischemia [71], as well as to restore blood flow in ischemic tissues after myocardial infarction [72]. Experimental data reported by Connor et al. indicate that the overexpression of manganese superoxide dismutase (SOD2) increases the production of mitochondrial H_2_O_2_, which triggers angiogenic activity. In this process, mitochondrial H_2_O_2_ can oxidize PTEN and upregulate the PI3K/AKT signaling axis, subsequently activating VEGF production [73] (Figure 1).

### 2.2. Vascular Constriction

Accumulating evidence highlights the significant role of PI3K/AKT-dependent signaling pathways in various fundamental cellular functions within the cardiovascular system. These functions include processes such as the maturation and growth, mechanotransduction, contractility, and proliferation and migration of both cardiac and vascular smooth muscle cells [74,75,76,77,78]. Dysfunction of this signaling pathway plays an essential role in cardiovascular pathophysiological conditions, such as heart failure, atherosclerosis, and hypertension [79,80,81,82]. Wu et al. observed that in the rostral ventrolateral medulla of spontaneously hypertensive rats, ROS originating from NOXs and mitochondrial oxidative stress reduced the catalytic ability of PTEN via oxidation. Consequently, the ensuing activation of the PI3K/AKT signaling pathway may lead to neurogenic hypertension [82].

Maintaining a consistent cerebral blood flow distribution through myogenic tone development is vital for neurons, which lack glucose storage and rely solely on a continuous blood supply of glucose and oxygen for normal metabolic function and under conditions of increased demands [83]. The role of PI3K in mediating the impact of physical forces, such as pressure, shearing, and stretching, on vascular smooth muscle cells and various other cell types, is well recognized [84]. Gebremedhin et al. found that elevated intraluminal pressure in cerebral arteries leads to an increase in ROS generation, leading to the oxidative inactivation of PTEN. This, in turn, results in the upregulation of PI3K/AKT activity and the release of IP3. The activation of AKT can induce the inhibition of arterial calcium-activated potassium channels, membrane depolarization, and L-type calcium channels. In addition, the formation of inositol (3,4,5)-triphosphate (IP3) stimulates the sarcoplasmic reticulum to release Ca^2+^, resulting in an increase in intracellular Ca^2+^ levels and the initiation of pressure-dependent myogenic constriction in cerebral arteries [83] (Figure 1).

### 2.3. Neuro-Regeneration and Neuro-Survival

PTEN activity has been shown to substantially limit cell survival in the challenging context of cerebral ischemia [64,65,66,67,68,69,70,71,72,73,74,75,76,77,78,79,80,81,82,83,84,85]. Numerous studies have demonstrated that inhibiting PTEN to activate the PI3K/AKT pathway provides protection to the brain during stroke [86,87,88,89,90,91]. The reduction in the PI3K/AKT/GSK-3β/mTOR signaling pathway by neuronal PTEN impairs axon growth and nerve regeneration in both the peripheral and central nervous systems, post-neuronal injuries, and ischemic conditions. Strong evidence consistently supports PTEN’s inhibitory role in critical neurological processes in pathological contexts [92,93,94,95,96,97,98]. Enhancing the activity of the PI3K/AKT pathway has been shown to increase axon growth [99]. Therefore, it is clear that PTEN, an intrinsic inhibitor of the PI3K pathway, plays a significant role in regulating the growth of central axons. PTEN’s activity also impedes nerve regeneration following neuronal injury, which is crucial for neural function recovery [96]. Hence, deliberately inhibiting PTEN activity emerges as a strategically advantageous approach with pronounced benefits for facilitating neuronal regeneration following injury. Empirical evidence shows that deleting PTEN in the spinal cord or optic nerve significantly enhances nerve regeneration after injury [100]. Targeted application of local pharmacological agents to suppress PTEN or the precise utilization of siRNA-based techniques to specifically downregulate PTEN expression at injury sites serves as a potent and effective strategy for accelerating the intricate axon outgrowth process and expediting the overall neuronal recovery [101]. Even in genetic diseases, such as spinal muscular atrophy, managing protein synthesis in motor neurons via PTEN depletion could be a therapeutic strategy [102,103]. Experimental data demonstrate that ROS signaling plays an essential role in promoting the self-renewal, proliferation, and differentiation of neural stem cells and neural progenitor cells via a regulatory mechanism in which the oxidation of PTEN by ROS upregulates the PI3K/AKT signaling pathway [104]. 

After neuronal injury, the injured axons are exposed to a highly oxidative and inflammation-driven environment. Under these conditions, growth cones, which are crucial for axon extension, initially collapse and retract. This process involves the oxidation of actin and produces ROS [105]. In a study, two experimental models were used to investigate the role of ROS generation in neuronal death and the involvement of PTEN in neurodegenerative diseases. Oxygen–glucose deprivation and the neurotoxin 1-methyl-4-phenylpyridinium iodide were applied to neural cells to simulate cerebral ischemia and Parkinson’s disease. However, it was found that ROS generated under these conditions did not cause oxidative inactivation to all cellular PTEN, allowing PTEN to maintain its functional activity. The suggested explanation is that the deactivation of PTEN phosphatase by ROS requires suitable intracellular co-localization with the site where these ROS are actively produced [106].

Hervera et al. have shown that non-mitochondrial sources of ROS are essential and sufficient for promoting axonal outgrowth and regenerating sensory axons. ROS signaling plays a crucial role in driving the regeneration of both peripheral and central nervous system axons in response to sciatic nerve injury. Importantly, NOX signaling emerges as a key regulatory mechanism in response to injury, particularly in ROS-dependent neuron regeneration. Membrane-bound NOX enzymes generate O_2_^•−^, which is subsequently converted to H_2_O_2_ by SOD. Interestingly, NOX2 can originate from extracellular vesicles released by cytokine-recruited inflammatory macrophages. These NOX2-containing exosomes are then transported retrogradely in axonal endosomes post-injury and produce ROS for cellular signaling. In other words, macrophages release NOX2-containing exosomes that subsequently enter the neurons and produce ROS, serving as a regeneration signal. These pathways involve key regulatory proteins whose activity can be modulated via the oxidation of cysteine residues. PTEN, notably, emerges as the most oxidized protein in such neurons following sciatic nerve injury. The downregulation of PTEN, mediated by NOX2 activity in association with nerve injury, leads to increased activation of the PI3K/AKT pathway, promoting neuron outgrowth. The PTEN oxidative inactivation following nerve injury plays an important role in regulating nerve regeneration and is, therefore, a prospective mechanism in the study of neuronal pathology [107].

In Alzheimer’s disease (AD), the accumulation of misfolded, hyperphosphorylated tau proteins is closely associated with the loss of neurons and cognitive dysfunction [108]. Tau normally plays a crucial role in assembling and maintaining microtubules in neuronal axons [109]. Abnormal hyperphosphorylation of tau alters its shape and impairs its ability to bind to microtubules, resulting in the destabilization of microtubules and the formation of neurofibrillary tangles, which contribute to neuronal dysfunction and cell death [110]. GSK-3β, a downstream kinase of the PI3K/AKT signaling pathway, is known for its role in phosphorylating tau in AD pathogenesis [111]. The impaired PI3K/AKT pathway leads to GSK-3β hyperactivity and excessive tau phosphorylation, which is linked to the progression of AD [112]. Treatment with insulin or curcumin can improve memory and cognitive ability in AD patients, possibly through the regulation of the PI3K/AKT pathway [113]. Stimulation with growth factors such as epidermal growth factor, platelet-derived growth factor, or insulin, leads to the formation of H_2_O_2_ as a result of the activation of NOXs and the oxidation of PTEN, which increases the PI3K/AKT signaling pathway [114]. These findings indicate that the oxidative inhibition of PTEN can be a possible method for improving AD patients’ condition.

**Figure 2:**
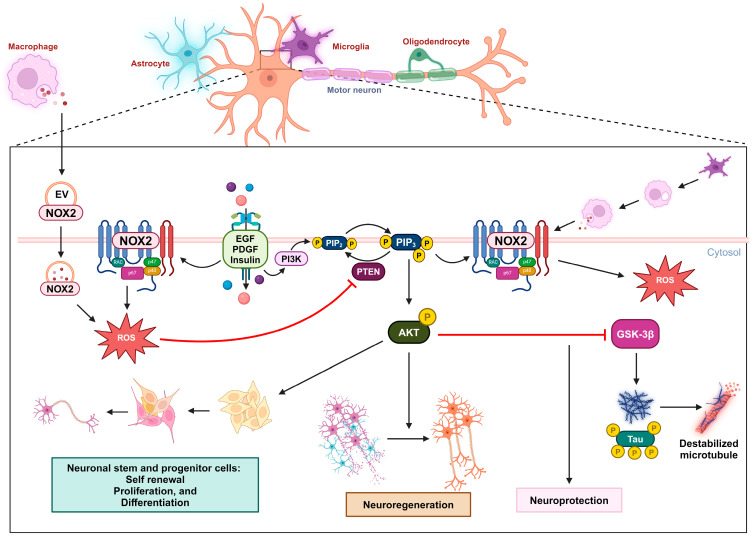
Oxidative inactivation of PTEN in nerve survival and regeneration. During neuronal injury, the NOX2-derived ROS concentration increases due to receptor kinase stimulation or extracellular vesicles released by macrophages. These ROS oxidize PTEN, leading to the activation of the PIP3/AKT signaling pathway, which promotes nerve regeneration. This mechanism can also promote self-renewal, proliferation, and differentiation in neuronal stem and progenitor cells. In the context of Alzheimer’s disease, the activation of the AKT pathway can downregulate GSK3β activity and the subsequent phosphorylation of the tau protein, providing neuroprotection.

Experimental data demonstrate that the presence of peroxynitrite can prevent etoposide-induced apoptotic cell death in primary cortical neurons. This effect is primarily due to the oxidation of PTEN and the subsequent upregulation of the PI3K/AKT signaling pathway. Although the anti-apoptotic implication of peroxynitrite is subject to dispute, these data concurrently strengthen the potential of PTEN oxidation in promoting neuroprotection [115] (Figure 2).

### 2.4. Immune Responsiveness

**Figure 3:**
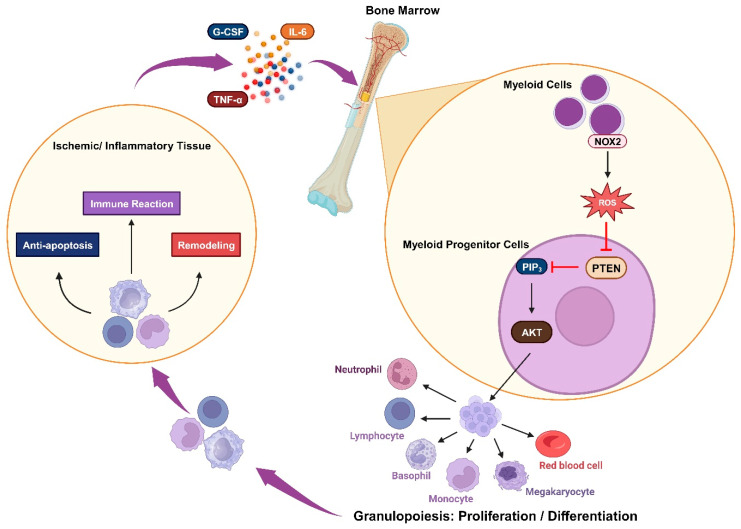
Oxidative inactivation of PTEN in immune responsiveness: Ischemia or inflammation can lead to elevated plasma cytokines, which stimulate myeloid cells to produce NOX2-derived ROS. These ROS mediate the AKT signaling pathway by inhibiting PTEN and trigger granulopoiesis, promoting the proliferation and differentiation of immune cells. These cells engage in immune reactions while also contributing to anti-apoptosis and remodeling processes.

Granulopoiesis is an emergency response to acute infection or inflammation, in which neutrophils are rapidly and massively produced and deployed from the bone marrow. Cytokines such as IL-6 and granulocyte colony-stimulating factor (G-CSF) are usually elevated during acute inflammation and may play a role in emergency granulopoiesis by inducing granulocyte differentiation [116,117]. In acute myocardial infarction, the myocardium also releases IL-6 and TNFα, and plasma levels of these cytokines increase after a brief episode of coronary artery blockage [118,119,120]. Kwak et al. demonstrated that an increase in ROS levels in the bone marrow alone is sufficient to trigger granulopoiesis. The elevated ROS concentration is important in promoting the proliferation and differentiation of myeloid progenitor cells via upregulated AKT signal transduction, which occurs due to the oxidative inhibition of PTEN’s phosphatase activity. During emergency granulopoiesis, these ROS are mainly produced by myeloid cells via phagocytic NOX2 activity, which can be induced by the cytokines G-CSF and TNFα. Therefore, the oxidative inactivation of PTEN by NADPH-oxidase-dependent ROS is an essential mechanism for prompting emergency granulopoiesis [121]. PI3K/AKT activity has also been shown to be a robust pivotal factor in the development of ROS-producing macrophages [122] (Figure 3).

### 2.5. Insulin-Related Metabolism

**Figure 4:**
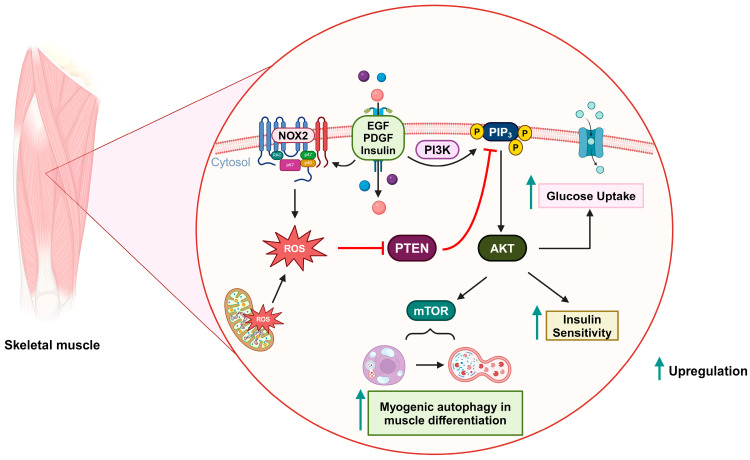
Oxidative inactivation of PTEN in insulin-related metabolism and muscle differentiation. Stimulation of growth factor receptors induces NOX2 activity and the production of ROS, which can oxidize PTEN and upregulate the PI3K/AKT signaling pathway. As a result, glucose uptake and insulin sensitivity are increased. During muscle differentiation, mitochondria-derived ROS can also oxidize PTEN and promote mTOR-induced myogenic autophagy.

Insulin resistance, which is characterized by a reduced sensitivity to insulin in regulating blood glucose levels, is the primary pathological feature of type 2 diabetes mellitus. The role of ROS in insulin sensitivity is complex, with a dual effect: promoting insulin sensitivity in the early stages of disease, and contributing to insulin resistance as hyperglycemia progresses. The transient and controlled ROS production by NOXs in response to insulin is likely to be beneficial, while the chronic ROS generation by mitochondria during the context of prolonged nutrient overload in the later stages of the disease might be detrimental to insulin responsiveness [123,124]. Insulin stimulation can lead to this temporary increase in ROS levels by activating NOX and subsequently triggering insulin-mediated AKT activation. PIP3 and NOXs are located in the cell’s plasma membrane, suggesting that upon insulin stimulation, PTEN is oxidatively inactivated in close proximity to NOXs, and recruited PI3K can elevate PIP3 levels [125]. PIP3, in turn, triggers the PDK/AKT pathway, which subsequently phosphorylates various targets such as AS160, performing the anabolic effects of insulin stimulation [126,127]. The activated AKT pathway can enhance glucose absorption in adipocytes by facilitating the translocation of glucose transporter GLUT4 to the plasma membrane, as well as elevating GLUT1 expression. This aligns with the proposition that AKT signaling potentially participates in mediating insulin-stimulated responses [128]. Hence, as a negative regulator of the AKT pathway, the knockout of PTEN was experimentally shown to incrementally affect the level of GLUT4 expression in skeletal muscle and white adipose tissue, which consequently increases glucose uptake [129,130]. Additionally, in some studies, inhibiting PTEN’s PIP3-phosphatase activity has been proposed as a potential therapeutic approach for type 2 diabetes [131,132,133]. Loh et al. demonstrated that a slight increase in physiological ROS levels in muscle cells can induce PTEN oxidation and eventually enhance insulin-induced glucose uptake via the PI3K/AKT pathway [124]. Therefore, the redox regulation of PTEN holds promise as a method for managing type 2 diabetes mellitus (Figure 4).

### 2.6. Myogenic Autophagy in Muscle Differentiation

Autophagy is a crucial intracellular recycling process that eliminates old and dysfunctional cellular proteins and organelles. This process involves the formation of autophagosomes, which envelop parts of the cell’s cytoplasm that contain unnecessary components. As a result, autophagy functions as a dynamic mechanism for maintaining cellular health and resource efficiency [134,135]. Kim et al. demonstrated that the PI3K/AKT/mTOR signaling pathway is upregulated by mitochondrial ROS-derived H_2_O_2_, which subsequently implicates myogenesis-specific autophagy during muscle differentiation. In this scenario, PTEN is inactivated via oxidation [136] (Figure 4).

## 3. Role of Bicarbonate in the Oxidation of PTPs by H_2_O_2_

H_2_O_2_ serves as a signaling molecule that participates in cellular responses triggered by various factors such as growth factors, hormones, and cytokines, including platelet-derived growth factor, epidermal growth factor, VEGF, insulin, TNFα, and interleukin-1β. During signal transduction, PTPs are key targets of growth-factor-mediated H_2_O_2_. These PTPs play a significant role in regulating multiple critical signaling pathways in mammalian cells by catalyzing the removal of phosphate groups from specific tyrosine residues on target proteins [32,137,138,139]. PTEN, which belongs to the PTP family and possesses the ability to dephosphorylate PIP3, can also be inactivated by physiological H_2_O_2_ [35].

The activation of receptor tyrosine kinases is a crucial event in the transmission of phosphorylation signals in response to growth factor stimulation, and it holds significant physiological importance [138]. When receptor tyrosine kinases are activated, they trigger the transient production of H_2_O_2_ by membrane NOXs [34]. This H_2_O_2_, in turn, leads to the reversible oxidative inhibition of PTPs [140]. However, the process by which PTPs undergo oxidation within the cellular environment has raised questions, particularly because other thiol proteins from the peroxiredoxin family are more significantly reactive and likely to interact with intracellular H_2_O_2_. In addition, oxidized PTPs, including PTEN, and peroxiredoxin can be converted back to their active reduced forms by the Trx/TrxR/NADPH systems, which are abundantly expressed in cells. 

H_2_O_2_ can react with bicarbonate/CO_2_ to form peroxymonocarbonate (HCO_4_^−^), a highly reactive oxidant that has a much higher reactivity than H_2_O_2_ when reacting with low-molecular-weight thiols [141,142]. Zhou et al. demonstrated that the presence of bicarbonate augments the oxidative inactivation of PTPs, particularly PTP1B and SHP-2, caused by H_2_O_2_, probably by generating HCO_4_^−^ [140]. Growth factor receptor stimulation also upregulates the activity of sodium bicarbonate cotransporters (NBCs) and carbonic anhydrase (CA) to increase the cellular concentration of bicarbonate. CA IX, a transmembrane enzyme with an extracellular active domain, can catalyze the following reaction: CO_2_ + H_2_O⇋HCO_3_^−^ [143]. NBCs uptake bicarbonate into the cell [144]. Via this mechanism, Dagnell et al. provide an explanation for the growth-factor-receptor-stimulation-mediated oxidation of PTP1B: with the increased level of bicarbonate, more HCO_4_^−^ is formed from H_2_O_2_, boosting the oxidative reaction rate [48]. Since PTEN’s molecular structure contains a cysteine residue in its active site, like other PTPs, the H_2_O_2_-mediated oxidative inhibition of PTEN can be affected by bicarbonate. In the future, further experiments should be conducted to fortify the role of bicarbonate in the redox regulation of PTEN by H_2_O_2_.

## 4. Conclusions and Perspectives

**Figure 5:**
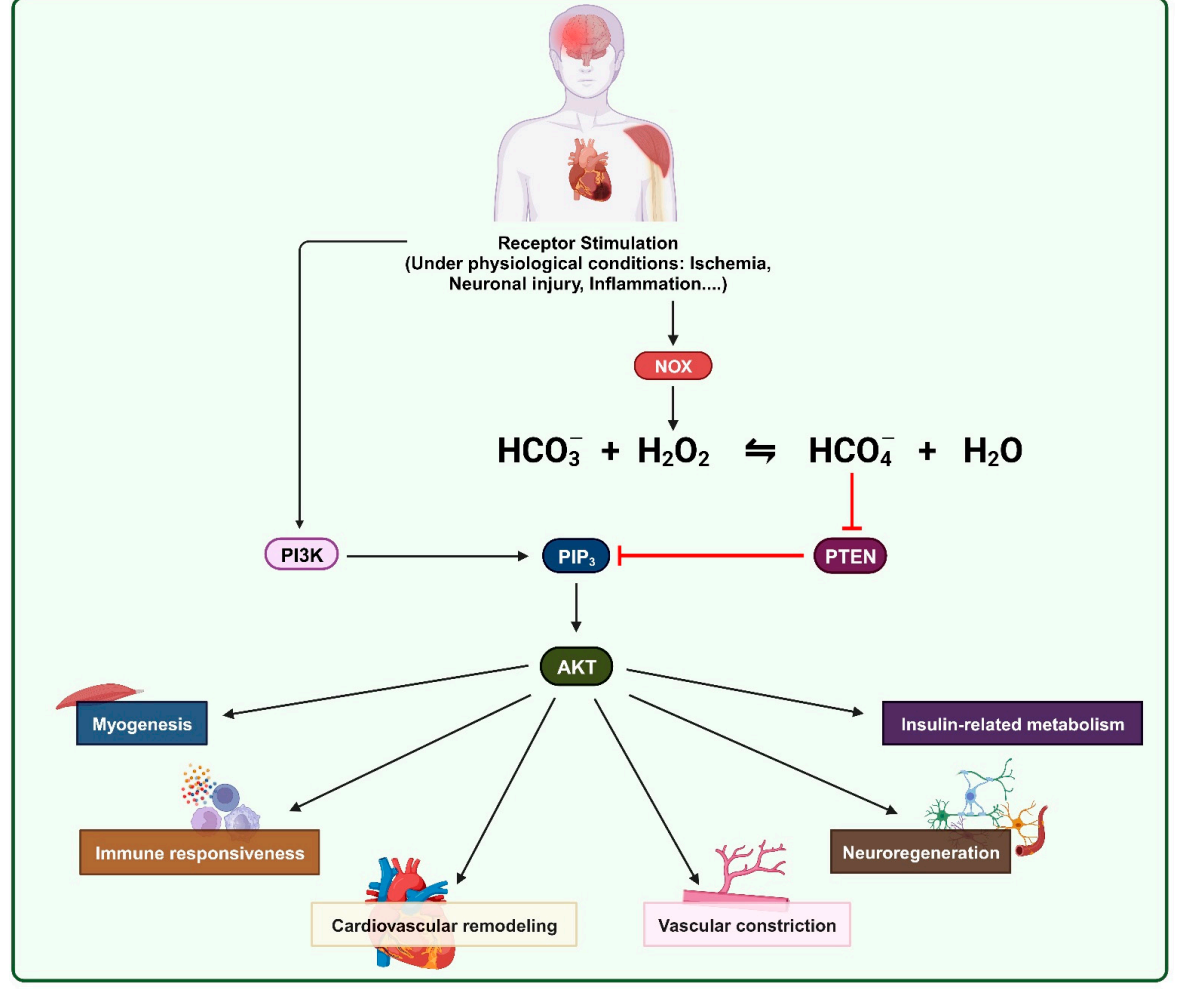
Stimulation of receptor tyrosine kinases can trigger the PI3K/AKT signaling pathway and promote H_2_O_2_ production via NOX2 activity. H_2_O_2_ can react with HCO_3_^−^ to form HCO_4_^−^ and inhibit PTEN, the negative regulator of the PI3K/AKT pathway. This mechanism plays a crucial role in physiological processes such as cardiovascular remodeling, vascular constriction, neuronal regeneration, immune responsiveness, insulin-related metabolism, and myogenesis.

In conclusion, PTEN oxidative inactivation by ROS, particularly NOX-derived H_2_O_2_, has been shown to be essential in various physiological processes, such as cardiovascular remodeling, vascular constriction, neuro-regeneration, immune responsiveness, insulin-related metabolism, and myogenesis-specific autophagy. This PTEN inactivation increases the activity of the PI3K/AKT signaling pathway and subsequently prevents apoptosis and promotes the proliferation of cardiomyocytes following ischemia, as well as increasing vascular angiogenesis and constriction. In the neuro-regeneration process, the ROS that oxidize PTEN could originate from the extracellular NOX2 delivery vesicles of macrophages. During acute ischemia or inflammation, ROS derived from NOX2 in myeloid cells can inhibit PTEN and induce granulopoiesis. The elevated PI3K/AKT downstream signaling via the redox regulation of PTEN could also mitigate insulin resistance. ROS also initiate cellular autophagic rebuilding in the process of muscle differentiation via PTEN-mediated mTOR augmentation. Moreover, bicarbonate can react with H_2_O_2_ to form HCO_4_^−^ and therefore accelerate the oxidation of PTPs. Further studies would substantiate the importance of HCO_4_^−^ in facilitating H_2_O_2_-mediated PTEN redox regulation and its role in physiological processes (Figure 5).

## Data Availability

Not applicable.
